# Prognostic significance of soft tissue deposits in laryngeal carcinoma^☆^

**DOI:** 10.1016/j.bjorl.2017.07.002

**Published:** 2017-07-31

**Authors:** Omer Afsin Ozmen, Melih Alpay, Ozlem Saraydaroglu, Uygar Levent Demir, Fikret Kasapoglu, Hamdi Hakan Coskun, Oguz Ibrahim Basut

**Affiliations:** a Uludag University Faculty of Medicine, Department of Otolaryngology, Bursa, Turkey; b Sanlıurfa Viransehir State Hospital, Sanlıurfa, Turkey; c Uludag University Faculty of Medicine, Department of Pathology, Bursa, Turkey

**Keywords:** Laryngeal cancer, Soft tissue deposit, Cervical metastasis, Prognosis, Survival, Câncer laríngeo, Depósito de tecido mole, Metástase cervical, Prognóstico, Sobrevida

## Abstract

**Introduction:**

Soft tissue deposits is tumorous islands apart from lymph nodes and occasionally diagnosed in neck dissection specimens. Their importance has begun to be recognized, however, their value has not been investigated in laryngeal cancer as a single tumor site.

**Objective:**

To investigate the prognostic value of soft tissue deposits in patients with laryngeal carcinoma.

**Methods:**

Medical records of 194 patients with laryngeal carcinoma who were treated primarily by surgery and neck dissection were reviewed. Prognostic significance of soft tissue deposits was assessed along with other clinical and pathological findings. Recurrence rates, overall and disease-specific survival rates were examined.

**Results:**

The incidence of soft tissue deposits was found to be 7.2% in laryngeal carcinoma. N stage was more advanced in patients who had soft tissue deposits. Regional recurrence rate was higher and disease specific and overall survivals rates were significantly lower in patients with soft tissue deposits in univariate analysis. However, in multivariate analysis, soft tissue deposits were not found as an independent risk factor.

**Conclusion:**

In laryngeal carcinoma, soft tissue deposits was diagnosed in patients with more advanced neck disease and their significance was lesser than other factors including extranodal extension.

## Resumo

**Introdução:**

Depósitos de tecido mole são ilhas tumorais diferente dos linfonodos e ocasionalmente diagnosticados em amostras de esvaziamento cervical. Sua importância começou a ser reconhecida, mas seu valor não foi investigado no câncer de laringe como um único local de tumor.

**Objetivo:**

Investigar o valor prognóstico do depósito de tecido mole em pacientes com carcinoma laríngeo.

**Método:**

Os prontuários de 194 pacientes com carcinoma laríngeo tratados principalmente por cirurgia e esvaziamento cervical foram analisados. O significado prognóstico dos depósitos de tecido mole foi avaliado juntamente com outros achados clínicos e histopatológicos. As taxas de recidiva, as taxas de sobrevida geral e específicas da doença foram avaliadas.

**Resultados:**

Observou-se uma incidência de depósitos de tecido mole de 7,2% no carcinoma laríngeo. O estágio N foi mais avançado em pacientes com depósitos de tecido mole. A taxa de recorrência regional foi maior e as taxas de sobrevida geral e específica da doença foram significativamente menores nesses pacientes na análise univariada. No entanto, na análise multivariada, o depósito de tecido mole não foi observado como um fator de risco independente.

**Conclusão:**

No carcinoma laríngeo, o depósito de tecido mole foi diagnosticado em pacientes com doença cervical mais avançada, mas sua significância foi menor do que outros fatores, incluindo a extensão extranodal.

## Introduction

Laryngeal carcinoma is the most common type of head and neck carcinomas excluding skin cancers.^1^ TNM staging is used for designating the optimal therapy and evaluating the prognosis of the patients. Among these factors, cervical metastases present as the most significant factor, decreasing 5 year survival rate by 50%.^2^ Factors related to cervical metastasis like extranodal extension (ENE), conglomerated lymph nodes and other tumor factors like differentiation, lymphovascular invasion (LVI) or perineural invasion (PNI), were also proposed to indicate prognostic significance, although, they were not included in the TNM staging.^3^, ^4^, ^5^

Violaris et al.^6^ first mentioned the term “soft tissue deposits (STD) or soft tissue free metastases” in the literature. Jose et al.^7^ defined soft tissue deposits as either extra-lymphatic squamous cell carcinoma (SCC) deposits or metastases that lacked lymphoid tissue due to total destruction of the lymph node architecture. Sarıoglu et al.^8^ indicated the absence of any sign of residual lymph node and localization in the lymphatic basin of primary tumor for the diagnosis of these tumor deposits.

Although pathological criteria of the STD were well established, they were not regularly reported in the studies and their clinical significance was not evident. The aim of the present study was to investigate the prognostic value of STD in patients with laryngeal carcinoma.

## Methods

### Study population

The present study was conducted in a university hospital, department of otolaryngology with the permission of local ethics committee (2014 – 23/11). A total of 269 patients who underwent neck dissection from January 1, 2007 to December 31, 2014 with the diagnosis of laryngeal carcinoma were retrospectively reviewed.

Patients who underwent simultaneous laryngectomy (partial or total) and neck dissection due to laryngeal SCC as the primary treatment were included in the analysis. Patients who were having surgery as salvage treatment (34 patients) or who did not have simultaneous laryngectomy and neck dissection (25 patients) were excluded, as were cases with synchronous tumors (3 patients) or who were lost to follow-up (13 patients). A follow-up period of at least one year was sought except for patients who had deceased prior to the completion of one postoperative year.

### Pathologic techniques and soft tissue deposits

Neck dissection materials, which were separated into zones peroperatively, were saturated in formalin for 24 h. Nodular lesions were separated (macroscopic pathological dissection) and embedded in paraffin blocks individually. These blocks were sectioned in their entirety at 4 μm thickness and sections were stained with H&E.

**Figure 1:**
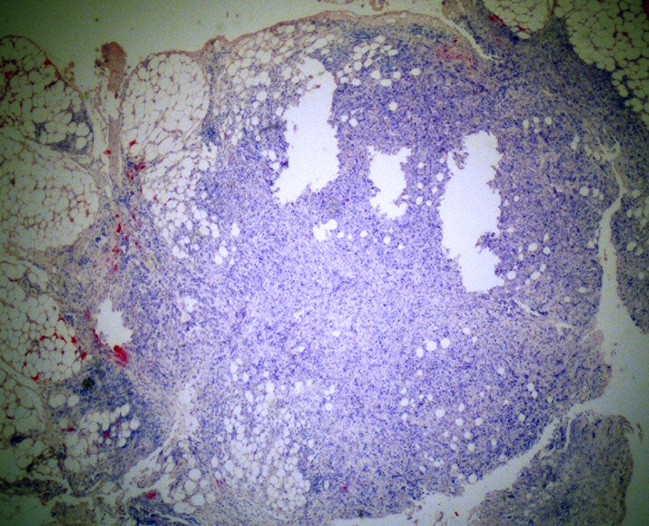
Histologic section of the soft tissue deposit. Irregular nodular mass composed of malign tumor cells infiltrating the lipomatous tissue is observed (H&E, ×40).

Any tumoral mass in the soft tissues of the neck dissection materials, with or without regular contours that did not have lymph node architecture were diagnosed as STD (Fig. 1).

### Outcome measures

The primary evaluation was the impact of STDs on oncologic outcome, which was represented by recurrence rate, overall and disease-specific survival rates.

### Other prognostic variables

Demographical (age, sex, smoking, alcohol consumption), clinical (comorbid diseases, preoperative tracheotomy) and pathological variables (histopathology and differentiation of tumor, LVI, PNI, surgical margins, ENE, conglomerated lymph nodes, STD) were analyzed according to free tumor implants and investigated as prognostic factors. TNM stage was calculated according to American Joint Committee on Cancer (AJCC) 2007 staging system.^9^

### Survival

The follow-up and status of patients were established from hospital records. The adherence to adjuvant treatment, complications, local/regional recurrences, distant metastasis, second primaries and cause of death was identified accordingly. The patients were grouped according to their final status as No Evidence of Disease (NED), Alive With Disease (AWD), Dead from Another Cause (DAC) and Dead Of Disease (DOD). Deaths due to postoperative complications were accepted as DAC.

### Statistical analysis

Analyses were performed with SPSS v.22.0 (IBM, NY, USA). Chi Square test was employed for categorical variables, Mann–Whitney-*U* test employed for numerical variables that did not show normal distribution. Kaplan–Meier survival curves were used to calculate 2 and 5 year disease-specific and overall survival estimates. The Kaplan–Meier estimates were compared using log-rank test to signify the effect of factors on survival individually. Cox proportional hazards regression model was used to calculate survival hazard ratios and 95% CIs. The level of statistical significance was defined as *p* < 0.05.

## Results

### Demographic and clinicopathologic characteristics

Of the 194 patients with laryngeal carcinoma included in the study, 189 were men (97.4%) and 5 were women (2.6%). The mean age of patients was 60.8 ± 8.6 with a range of 41–84 years.

Demographical, clinical and pathological variables were analyzed according to presence or absence of free tumor implants (Table 1). N stage was more advanced and ENE, conglomerated lymph nodes, PNI and LVI were more frequent in patients who had STD compared to those who did not.

**Table 1:** Demographical, clinical and pathological factors and their distribution according to soft tissue deposits.

	Soft tissue deposit		Total *n* (%)	*p*-Value
	Present *n* (%)	Absent *n* (%)		
*Sex*				1
Male	14 (100)	175 (97.2)	189 (97.4)	
Female	0 (0)	5 (2.8)	5 (2.6)	

*Smoking*				1
Yes	12 (85.7)	157 (87.2)	169 (87.1)	
No	2 (14.3)	23 (12.8)	25 (12.9)	

*Comorbid disease*				1
Present	5 (35.7)	62 (34.4)	67 (34.5)	
Absent	9 (64.3)	118 (65.6)	127 (65.5)	

*Preoperative tracheotomy*				0.085
Yes	7 (50)	45 (25)	52 (26.8)	
No	7 (50)	135 (75)	142 (73.2)	

*Histopathological type*				0.546
Classical SCC	14 (100)	156 (86.7)	170 (87.6)	
Basaloid SCC	0 (0)	18 (10)	18 (9.3)	
Verrucous SCC	0 (0)	1 (0.6)	1 (0.5)	
Papillary SCC	0 (0)	5 (2.8)	5 (2.6)	

*Differentiation*				0.589
Well	1 (7.1)	23 (13.1)	24 (12.6)	
Moderately	7 (50)	99 (56.2)	106 (55.8)	
Poorly	6 (42.9)	54 (30.7)	60 (31.6)	

*Pathological stage*				0.071
Stage 1	0 (0)	9 (5)	9 (4.6)	
Stage 2	0 (0)	26 (14,4)	26 (13.4)	
Stage 3	1 (7.1)	42 (23.3)	43 (22.2)	
Stage 4	13 (92.9)	103 (57.2)	116 (59.8)	

*T stage (pathological)*				0.593
T1	1 (7.1)	9 (5.0)	10 (5.2)	
T2	1 (7.1)	37(20.6)	38 (19.6)	
T3	4 (28.6)	51 (28.3)	55 (28.4)	
T4	8 (57.1)	83 (46.1)	91 (46.9)	

*N stage (pathological)*				<0.001
N0	0 (0)	109 (60.6)	109 (56.2)	
N1	2 (14.3)	36 (20)	38 (19.6)	
N2	10 (71.4)	33 (18.3)	43 (22.2)	
N3	2 (14.3)	2 (1.1)	4 (2.1)	

*Surgical margin*				1
Negative	14 (100)	176 (97.8)	190 (97.9)	
Positive	0 (0)	4 (2.2)	4 (2.1)	

*Extracapsular spread*				0.002
Present	6 (42.9)	19 (10.6)	25 (12.9)	
Absent	8 (57.1)	161 (89.4)	169 (87.1)	

*Conglomerated lymh nodes*				<0.001
Present	8 (57.1)	11(6.1)	19 (9.8)	
Absent	6 (42.9)	169 (93.9)	175 (90.2)	

*Perineural invasion*				0.003
Present	8 (57.1)	34 (19.3)	42 (22.1)	
Absent	6 (42.9)	142 (80.7)	148 (77.9)	

*Lymphovascular invasion*				<0.001
Present	5 (35.7)	8 (4.5)	13 (6.8)	
Absent	9 (64.3)	168 (95.5)	177 (93.2)	

*Total*	14 (7.2)	180 (92.8)	194 (100)	

Type of surgery and utilization of adjuvant treatment were similar in respect of STD whereas, STD was found more frequently in the comprehensive neck specimens (Table 2).

**Table 2:** Treatment modalitities employed in patients and their distribution according to soft tissue deposits.

	Soft tissue deposit		Total *n* (%)	*p*-Value
	Present *n* (%)	Absent *n* (%)		
*Laryngectomy*				0.399
Partial	4 (28.6)	72 (40)	76 (39.2)	
Total	10 (71.4)	108 (60)	118 (60.8)	

*Neck dissection*				<0.001
Selective	3 (21.4)	168 (93.3)	171 (88.1)	
Comprehensive	11 (78.6)	12 (6.7)	23 (11.9)	

*Adjuvant treatment*				0.204
Radiotherapy	6 (42.9)	75 (41.7)	81 (41.8)	
Chemoradiotherapy	6 (42.9)	45 (25)	51 (26.3)	
None	2 (14.3)	60 (33.3)	62 (32.0)	

*Total*	14 (7.2)	180 (92.8)	194 (100)	

### Oncological outcome and survival

The mean follow-up duration was 36.6 ± 23.4 months. Ten patients (5.2%) developed local and 16 (8.2%) developed regional recurrences. Two patients had both local and regional recurrences thus; the overall loco-regional recurrence rate was established as 12.4%. Distant metastases were diagnosed in 15 patients (7.7%). Second primary tumors appeared in 24 patients (12.4%) during follow-up. Regional recurrences were found to be more common in patients with STD (Table 3).

**Table 3:** Oncologic occurrences in the follow-up and their distribution according to soft tissue deposits.

	Soft tissue deposit		Total *n* (%)	*p*-Value
	Present *n* (%)	Absent *n* (%)		
*Local recurrence*				
Present	0 (0)	10 (5.5)	10 (5.2)	0.781
Absent	14 (100)	170 (94.4)	184 (94.8)	

*Regional recurrence*				
Present	4 (28.6)	12 (6.7)	16 (8.2)	0.018
Absent	10 (71.4)	168 (93.3)	178 (91.8)	

*Distant metastasis*				
Present	2 (14.3)	13 (7.2)	15 (7.7)	0.664
Absent	12 (85.7)	167 (92.8)	179 (92.3)	

*Second primary*				
Present	2 (14.3)	22 (12.2)	24 (12.4)	1
Absent	12 (85.7)	158 (87.8)	170 (87.6)	

*Total*	14 (7.2)	180 (92.8)	194 (100)	

Final status of 131 patients (67.5%) was NED, 9 (4.6%) AWD, 25 (12.9%) DOD, 22 (11.3%) DAC, and 7 (3.6%) were lost due to perioperative complications that were also accepted as DAC.

Disease specific survival was 79.8 ± 2.3 and overall survival was 68.1 ± 2.8 months for the whole study group according to Kaplan–Meier estimates.

**Figure 2:**
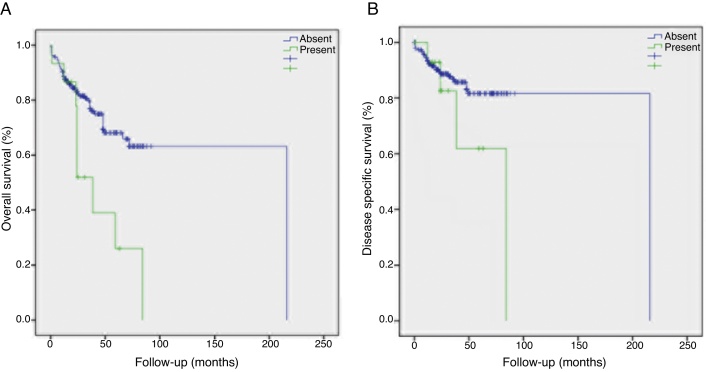
Kaplan–Meier plots demonstrating (A) overall and (B) disease-specific survival rates by soft tissue deposits.

Disease specific survival was 45.1 ± 6.9 months for patients with STD and 81.1 ± 2.2 months for those without STD. Two and 5 years disease specific survival rates were 90.3% and 84.8% in patients without STD and 74% and 49.4% respectively in patients with STD (Table 4, Fig. 2). STD was found to have a significant influence on disease specific survival (*p* = 0.041).

**Table 4:** Two and 5 years disease specific and overall survival rates of the patients according to clinical and pathological factors.

	Disease specific survival rate (%)			Overall survival rate (%)		
	2 years	5 years	*p*-Value	2 years	5 years	*p*-Value
*Smoking*						0.041
Yes	87.5	80.2	0.044	79.9	64.6	
No	100	100		95.7	79.7	

*Comorbid disease*						0.076
Absent	89.5	81	0.686	85.1	74.6	
Present	88.4	85.6		76.2	57.1	

*Preoperative tracheotomy*						0.67
Absent	90.5	84.5	0.206	84.1	71.5	
Present	85.2	78.3		75.3	55.1	

*Tumor type*						0.534
Classical	89.1	81.8	0.508	81.1	63.3	
Basaloid	100	88.9		87.7	78.0	
Papillary	100	100		82.4	66.7	

*Differentiation*						0.955
Well	90.9	83.3	0.894	83.1	65.3	
Moderate	89.0	80.5		81.9	60.7	
Poor	91.8	85.8		78.2	67.1	

*Surgical margin*						0.141
Negative	89.9	83.5	0.003	82.5	67.5	
Positive	50	50		50	50	

*N stage*						0.004
0	94.9	90.2	0.017	91.4	75.3	
1	86	79.4		75.9	65.9	
2	74.7	67.9		59.7	46.5	
3	50	50		50	50	

*Soft tissue deposits*						0.002
Present	74	49.4	0.041	78.6	16.4	
Absent	90.3	84.8		84.3	71.2	

*Extracapsular spread*						<0.001
Present	59.1	50.7	< 0.001	56.8	37.9	
Absent	92.9	86.9		86.9	71.2	

*Conglomerated lymph nodes*						0.025
Present	74.6	74.6	0.143	55.8	37.2	
Absent	90.5	83.8		84.4	70.1	

*Perineural invasion*						0.002
Present	82.7	72.3	0.105	67.6	45.5	
Absent	90.5	84.9		85.2	71.9	

*Lymphovascular invasion*						0.167
Present	92.3	76.9	0.848	75.2	53.7	
Absent	88.6	82.9		82	67.7	

*Neck dissection type*						<0.001
Selective	90.3	87.1	0.001	85.9	74.3	
Comprehensive	78.5	37.2		51.1	12.1	

*Local recurrences*						0.007
Present	50	40	<0.001	50	40	
Absent	91.7	86		83.8	68.9	

*Regional recurrences*						<0.001
Present	55	34.4	<0.001	55	24.1	
Absent	92.7	88		84.5	71.8	

*Distant metastasis*						<0.001
Present	51.4	27.4	<0.001	51.4	22.9	
Absent	92.4	87.5		84.3	70.9

Overall survival was 35.3 ± 6.2 months in patients with STD and 70.3 ± 2.8 months in patients free of STD. Two and 5 year overall survival rates were 84.3% and 71.2% when there was no evidence of STD in the specimen. On the other hand, it was to be 49.1% and 16.4% respectively in cases where STD was present. Overall survival rates were significantly lower in the presence of STD compared to its absence (*p* = 0.002).

T stage, preoperative tracheotomy, tumor type and differentiation, LVI and comorbid diseases did not affect the survival rates.

History of smoking, PNI, surgical margins for the laryngeal specimen, N stage, ENE, conglomerated lymph nodes and STD for the neck specimen, type of neck dissection, and recurrences or distant metastasis were found to have detrimental effects on survival (Table 4).

Significant factors from univariate analysis were tested with multivariate Cox regression analysis (Table 5). ENE was found to increase the risk of death from the laryngeal cancer 3.47 times. Positive surgical margin was also a significant factor for disease specific survival. Overall survival was significantly reduced by smoking and PNI. ENE was found to be related to overall survival, however, to a lesser extent.

**Table 5:** Factors affecting survival according to multivariate analysis and the hazard ratios.

	Hazard ratio (95% CI)	*p*-Value
*Disease specific survival*		
Extracapsular spread	3.470 (1.209–9.964)	0.021
Surgical margin	0.203 (0.043–0.965)	0.045

*Overall survival*		
Smoking	4.447 (1.058–18.687)	0.042
Extracapsular spread	2.197 (0.974–4.957)	0.058
Perineural invasion	2.166 (1.129–4.153)	0.02

## Discussion

Soft tissue deposits were reported as having an overall incidence of 17.1–24.3 in head and neck cancers.^7^, ^8^, ^10^ In subgroup analysis, larynx carcinoma was noticed to be associated with lesser STDs (14.3–16.1%) compared to oral cavity (20–22.6%) and hypopharynx cancers (22.7–38.5%).^8^, ^10^ The incidence of soft tissue deposits was 7.2% in the present study, which was lower than the literature. Exclusion of patients who underwent prior radiotherapy and pathological examination techniques might be associated with lower STD rates than the literature.

STDs were observed to be more frequent in patients with advanced neck disease, which is confirmed by previous studies.^7^, ^8^ Five percent of patients with N1, 23% with N2 and 50% with N3 neck tumors had STD, whereas, none of the patients with N0 neck tumors were found to have STD. Sarıoglu et al.^8^ reported a single case of STD in a pathologically N0 neck.

ENE, which also indicated advanced neck disease, was associated with STD more commonly. Forty-three percent of the STDs were found together with ENE, whereas 24% of ENEs were coexisting with STD. This was also concordant with the literature.^7^, ^8^, ^10^

Other prognostic factors like conglomerated lymph nodes, PNI and LVI were found to be associated with STDs, which indicated more aggressive tumor behavior, however, certain factors indicating tumor aggressiveness such as histopathological subtype of the tumor or tumor differentiation were not associated with STD.

Soft tissue deposits might represent true extra-nodal metastases or nodal metastases in which all nodal architecture was lost due to tumor infiltration.^10^ Either aggressive tumor behavior or poor host defense might be the underlying cause. Either way, the process implies poor prognosis theoretically.

On clinical grounds, patients with STD experienced regional recurrences 4 times more than those without STD. STDs were reported to increase recurrence and distant metastasis rates 2.29 times in the literature.^8^ Survival period of patients with STD were about half of those without STD. In univariate analyses, STD was found to decrease survival rates significantly, however, in multivariate analysis, STD was not found to be an independent prognostic factor. In the literature, STDs were reported to be an independent factor for head and neck carcinomas that decrease overall survival by 3.2 times in one study.^8^ Relatively lower incidence of STD observed in laryngeal carcinoma might have led other potent prognostic factors to cover the importance of STD in our study. Nevertheless, STDs would seem to have a significant prognostic impact on laryngeal cancer outcome and we recommended STD to be included in the pathologic analysis and taken into consideration in the decision tree.

Multivariate analysis confirmed ENE, positive surgical margins and PNI as independent prognostic factors in laryngeal carcinoma. These factors had already been accepted as important prognostic factors and were targeted by the adjuvant treatments in many institutions.^11^, ^12^, ^13^, ^14^ Interestingly, smoking was found as an important risk factor affecting overall survival. Risk of second primary tumors or increase in complications and comorbid diseases might provide mechanisms for smoking to exert its effects.^15^

Even though they are all SCCs, head and neck carcinomas present diverse clinical characteristics. Thus, we believed the analysis of STD in laryngeal carcinoma only was valuable in identifying its prognostic significance.

## Conclusion

In laryngeal carcinoma, STD was diagnosed in patients with more advanced neck disease and it played a lesser significance than other factors including ENE. The retrospective nature of the study and relatively small study population suggests further studies are needed.

## Ethical approval

All procedures performed in studies involving human participants were in accordance with the ethical standards of the institutional research committee and with the 1964 Helsinki declaration and its later amendments or comparable ethical standards.

## Conflicts of interest

The authors declare no conflicts of interest.

## References

[1] Silverberg E., Boring C.C., Squires T.S. (1990). Cancer statistics 1990. CA Cancer J Clin.

[2] Hahn S.S., Spaulding C.A., Kim J.A., Constable W.C. (1987). The prognostic significance of lymph node involvement in pyriform sinus and supraglottic cancers. Int J Radiat Oncol Biol Phys.

[3] Gallo A., Manciocco V., Simonelli M., Pagliuca G., D’Arcangelo E., de Vincentiis M. (2005). Supracricoid partial laryngectomy in the treatment of laryngeal cancer: univariate and multivariate analysis of prognostic factors. Arch Otolaryngol Head Neck Surg.

[4] Zatterstrom U.K., Wennerberg J., Ewers S.B., Willen R., Attewell R. (1991). Prognostic factors in head and neck cancer: histologic grading, DNA ploidy, and nodal status. Head Neck.

[5] Stell P.M. (1988). Prognostic factors in laryngeal carcinoma. Clin Otolaryngol Allied Sci.

[6] Violaris N.S., O’Neil D., Helliwell T.R., Caslin A.W., Roland N.J., Jones A.S. (1994). Soft tissue cervical metastases of squamous carcinoma of the head and neck. Clin Otolaryngol Allied Sci.

[7] Jose J., Coatesworth A.P., Johnston C., MacLennan K. (2003). Cervical node metastases in squamous cell carcinoma of the upper aerodigestive tract: the significance of extracapsular spread and soft tissue deposits. Head Neck.

[8] Sarioglu S., Akbulut R.N., Iplikci S., Aydin B., Dogan E., Unlu M. (2016). Tumor deposits in head and neck carcinomas. Head Neck.

[9] Edge S.B., Byrd D.R., Compton C.C., Fritz A.G., Greene F.L., Trotti A. (2010). Larynx. AJCC cancer staging manual.

[10] Jose J., Moor J.W., Coatesworth A.P., Johnston C., MacLennan K. (2004). Soft tissue deposits in neck dissections of patients with head and neck squamous cell carcinoma: prospective analysis of prevalence, survival, and its implications. Arch Otolaryngol Head Neck Surg.

[11] Zhang S.Y., Lu Z.M., Luo X.N., Chen L.S., Ge P.J., Song X.H. (2013). Retrospective analysis of prognostic factors in 205 patients with laryngeal squamous cell carcinoma who underwent surgical treatment. PLoS ONE.

[12] Alvi A., Johnson J.T. (1996). Extracapsular spread in the clinically negative neck (N0): implications and outcome. Otolaryngol Head Neck Surg.

[13] Fagan J.J., Collins B., Barnes L., D’Amico F., Myers E.N., Johnson J.T. (1998). Perineural invasion in squamous cell carcinoma of the head and neck. Arch Otolaryngol Head Neck Surg.

[14] Yılmaz T., Hosal A.S., Gedikoğlu G., Önerci M., Gürsel B. (1998). Prognostic significance of vascular and perineural invasion in cancer of the larynx. Am J Otolaryngol.

[15] Wulff N.B., Kristensen C.A., Andersen E., Charabi B., Sørensen C.H., Homøe P. (2015). Risk factors for postoperative complications after total laryngectomy following radiotherapy or chemoradiation: a 10-year retrospective longitudinal study in Eastern Denmark. Clin Otolaryngol.

